# Retrospective Clinical Investigation into the Association Between Abnormal Blood Clotting, Oral Anticoagulant Therapy, and Medium-Term Mortality in a Cohort of COVID-19 Patients

**DOI:** 10.3390/biomedicines13030535

**Published:** 2025-02-20

**Authors:** Giorgia Dinoi, Maria Vittoria Togo, Pietro Guida, Caterina Deruvo, Francesco Samarelli, Paola Imbrici, Orazio Nicolotti, Annamaria De Luca, Franco Mastroianni, Antonella Liantonio, Cosimo Damiano Altomare

**Affiliations:** 1 Department of Pharmacy—Pharmaceutical Sciences, University of Bari Aldo Moro, 70125 Bari, Italy; giorgia.dinoi@uniba.it (G.D.); maria.togo@uniba.it (M.V.T.); caterina.deruvo@uniba.it (C.D.); francesco.samarelli@uniba.it (F.S.); paola.imbrici@uniba.it (P.I.); orazio.nicolotti@uniba.it (O.N.); annamaria.deluca@uniba.it (A.D.L.); 2 Department of Internal Medicine, F. Miulli General Hospital, 70021 Bari, Italy; p.guida@miulli.it (P.G.); f.mastroianni@miulli.it (F.M.)

**Keywords:** oral anticoagulant therapy, direct oral anticoagulants, vitamin K antagonists, aldosterone antagonists, SARS-CoV-2, COVID-19

## Abstract

**Background/Objectives:** People affected by COVID-19 are exposed to abnormal clotting and endothelial dysfunction, which may trigger thromboembolic events. This study aimed at retrospectively investigating whether oral anticoagulant therapy (OAT), encompassing either direct oral anticoagulants (DOACs), mainly apixaban, or the vitamin K antagonist (VKA) warfarin, could have impacted medium-term mortality in a cohort of SARS-CoV-2 patients. **Methods:** Among 1238 COVID-19 patients, hospitalized from 17 March 2020 to 15 June 2021, 247 survivors and 247 deceased within 90 days from hospitalization were matched 1:1 based on age, sex, and intensive care unit (ICU) admission within three days. Conditional logistic regression was used to estimate associations by means of odds ratio (OR) with a 95% confidence interval (CI). **Results:** A univariate regression analysis suggested that OAT, no differently from subcutaneous low-molecular-weight heparins (LMWHs) during hospitalization, has no significant impact (*p* value > 0.05) on medium-term mortality. A multivariate analysis, limited to baseline variables (i.e., comorbidities and pharmacotherapies at hospital admission) showing significant association (*p* < 0.05) to mortality in a univariate analysis, revealed that, compared to patients living at 90 days from hospitalization, deceased patients had cancer histories (OR 1.75, CI 1.06–2.90, *p* = 0.029) or suffered from asthma (OR 2.25, CI 1.13–4.47, *p* = 0.021). In contrast, heart failure (HF), atrial fibrillation (AF), arteriopathy, chronic obstructive pulmonary disease (COPD), and kidney failure (KF), which, in a univariate analysis, were found to be associated with the endpoint (*p* < 0.05), lost significance in a multivariate analysis. Therapy at admission with aldosterone antagonists also appeared to be associated with medium-term mortality (OR 2.49, CI 1.52–4.08, *p* < 0.001); whereas, vitamin D supplementation during hospitalization appeared to be beneficial. Although not conclusive, a search into the Eudravigilance database, combined with consulting a digital predictive platform (PLATO, polypharmacology platform prediction), suggested potential off-target activities, which might contribute to increasing the severity of SARS-CoV-2 infection. **Conclusions:** This retrospective clinical study furnished evidences of the impact of OAT, comorbidities and other pharmacological treatments on COVID-19 clinical course.

## 1. Introduction

Severe acute respiratory syndrome coronavirus 2 (SARS-CoV-2) is the human coronavirus identified as the cause of COVID-19. First detected in China in December 2019, the disease has rapidly spread worldwide, resulting in a global pandemic. COVID-19 clinical symptoms include fever, dry cough, sore throat, dyspnea, headache, severe asthenia, and interstitial pneumonia, which can potentially progress to alveolar damage and subsequent respiratory failure [1,2].

From the onset of the pandemic, apart from vaccines approved by the US Food and Drug Administration (FDA) and the EU European Medicine Agency (EMA) between December 2020 and January 2021, no specific small-molecule drug had been approved for the treatment of COVID-19 until December 2021 [3]. It was about two years after the COVID-19 pandemic onset that approval in US and UK (December 2021) and EU and Canada (January 2022) of Paxlovid was obtained. Paxlovid is an oral formulation of nirmatrelvir (an inhibitor of SARS-CoV-2 main protease) and ritonavir (a CYP3A inhibitor), which proved to be effective in decreasing the risk of severe COVID-19 or mortality [4]. Until the end of 2021, i.e., six months after the period of our observation (see below), investigational therapies were undergoing preclinical and clinical trials for COVID-19, including immunomodulators, antivirals, and antibiotics, such as, for example, remdesivir, ribavirin, hydroxychloroquine, azithromycin, nitazoxanide, and teicoplanin [5,6].

The high mortality rate associated with COVID-19 is not only a result of viral replication in lung epithelial cells but is also driven by a dysregulated host immune response defined as “cytokine storm syndrome” [7]. COVID-19 infection increases the risk of arterial and venous thrombosis, which has led to considerable interest in antithrombotic treatment to prevent and manage the related complications [8]. Attention has been paid to the repurposing of serine protease inhibitors, including direct oral anticoagulants (DOACs), such as the factor Xa-selective inhibitor otamixaban [9] and the thrombin-selective inhibitor nafamostat [10]. These treatments aimed at characterizing molecules capable of acting both at the level of virus entry (i.e., blocking the protease activation of the Spike protein by inhibiting TMPRSS2 transmembrane serine protease 2) and the downstream thrombotic complications.

**Figure 1:**
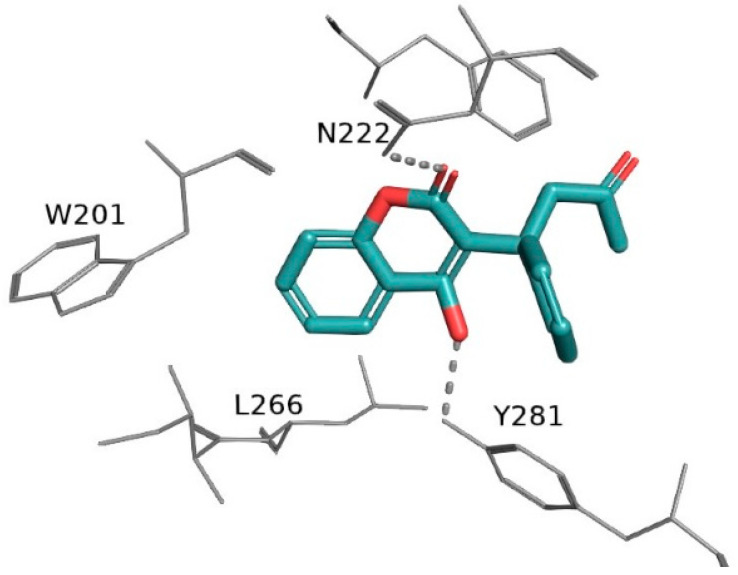
X-ray crystal structure of warfarin in complex with vitamin K epoxide reductase complex 1 (VKORC1, PDB ID: 6WV3). Only key amino acids are displayed. H-bonds and polar interactions are shown as dashed lines. The warfarin structure is rendered as sticks; whereas, key interacting residues are rendered as lines. The oxygen atoms are colored red; the hydrogen atoms are omitted for clarity.

An increasing number of studies highlighted abnormal serum coagulation parameters in hospitalized patients with severe forms of COVID-19, suggesting a tendency toward a hypercoagulable state. This state may result in a prevalent venous thromboembolism (VTE) especially in non-typical locations, disseminated intravascular coagulation (DIC), and ARDS [11]. In addition, endothelial damage causing microvascular pulmonary thrombosis has been associated with poor clinical outcomes in patients with interstitial pneumonia [12]. Despite the fast-growing understanding of the clinical features and natural history of infection of COVID-19, many aspects of the disease still remain unaddressed [13,14]. The high risk of COVID-19-associated thrombotic events and the related role of anticoagulants is one of the unresolved issues. Anticoagulant drugs, such as heparins, vitamin K antagonists (VKAs), and more recently, DOACs, are used in managing thrombotic disorders. VKAs, such as warfarin, were the preferred anticoagulant agents before the approval of DOACs. Warfarin inhibits the vitamin K epoxide reductase complex 1 (VKORC1, Figure 1), thereby reducing the synthesis of clotting factors II, VII, IX, and X.

**Figure 2:**
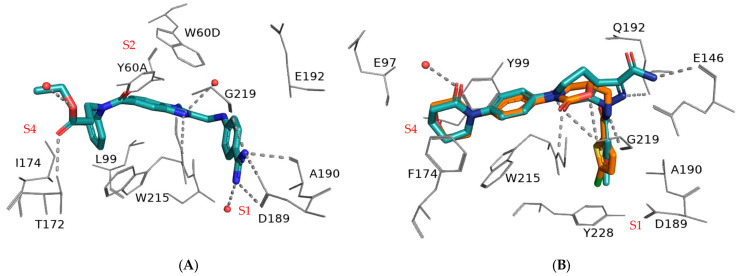
(**A**) X-ray crystal structures of dabigatran in complex with thrombin (PDB ID: 1KTS) and (**B**) superposition of x-ray crystal structures of rivaroxaban (orange, PDB ID: 2W26) and apixaban (cyan, PDB ID: 2P16) in complex with human factor Xa. Only key residues lining the main binding subsites S1, S2 and S4 are displayed. H-bonds and polar interactions are shown as dashed lines; water molecules within 4 Å from the ligand are displayed as red spheres. The ligands are rendered as tubes, and the key residues as lines. The nitrogen and oxygen atoms are colored in blue and red, respectively; the hydrogen atoms are omitted for clarity.

DOACs in clinical use proved to be more effective and safer than warfarin. They act as selective inhibitors of thrombin (factor IIa, e.g., dabigatran; Figure 2A) or activated factor X (factor Xa, e.g., apixaban and rivaroxaban in Figure 2B, and edoxaban) in the blood coagulation cascade. DOACs offer several advantages over warfarin, such as rapid onset, short duration of action, and reversible binding to their targets. Additionally, they simplify clinical management, especially by reducing the frequency of monitoring and risk of bleeding [15,16].

Given that early endothelial injury leading to microvascular pulmonary thrombosis may be associated with poor clinical outcomes in patients with respiratory failure caused by interstitial pneumonia, the hypothesis was put forward that COVID-19 patients receiving anticoagulant therapy at the time of the infection might be protected from adverse outcomes, as compared to non-treated patients. In October 2021, the International Society on Thrombosis and Haemostasis (ISTH) issued evidence-based recommendations concerning the use of anticoagulants and antiplatelet agents for patients diagnosed with COVID-19 across various clinical contexts [17].

Despite the plethora of studies on the management of COVID-19 that had been published, a substantial uncertainty persists regarding the use of anticoagulants for the prevention and treatment of thromboembolic events in hospitalized COVID-19 patients. In this context, some studies focused on patients already on oral anticoagulant therapy (OAT) when diagnosed with COVID-19, based on the hypothesis that they could be at lower risk of adverse outcomes. A large randomized controlled trial showed that treatment with anticoagulants reduced the risk of death in COVID-19 patients admitted to the intensive care unit (ICU) [18]. However, controversial findings have emerged regarding the role of OAT on diverse clinical outcomes of COVID-19 patients were reported. Some studies showed that prior use of OAT did not improve survival in hospitalized COVID-19 patients with similar outcomes for both patients treated with VKAs or DOACs [19,20]. In contrast, a retrospective cohort study reported that COVID-19 patients receiving OAT at the time of infection and during the disease showed significantly lower risk of all-cause mortality at 21 days [21]. Another study reported that, among elderly patients hospitalized for COVID-19, patients receiving OAT had a significantly lower mortality rate during hospitalization [22,23]. A retrospective observational study from CORIST registry also showed that OACs might have protective effects on adverse COVID-19 outcomes in hospitalized patients with atrial fibrillation (AF) and that fewer adverse events occurred in patients receiving therapy with DOACs compared to patients receiving therapy with VKAs [24]. Interestingly, another study reported that high-risk AF patients receiving OAT had a lower risk of receiving a positive COVID-19 test and severe COVID-19 outcomes [25].

In light of the above considerations, a major purpose of this retrospective study is to assess whether OAT (with either DOACs or VKAs) could impact the medium-term mortality of COVID-19 patients. To this end, a cohort of patients hospitalized for COVID-19 during pandemic was analyzed, focusing on the association of OAT with patients’ characteristics, disease severity, comorbidities, and comedications.

## 2. Materials and Methods

### 2.1. Study Population

**Figure 3:**
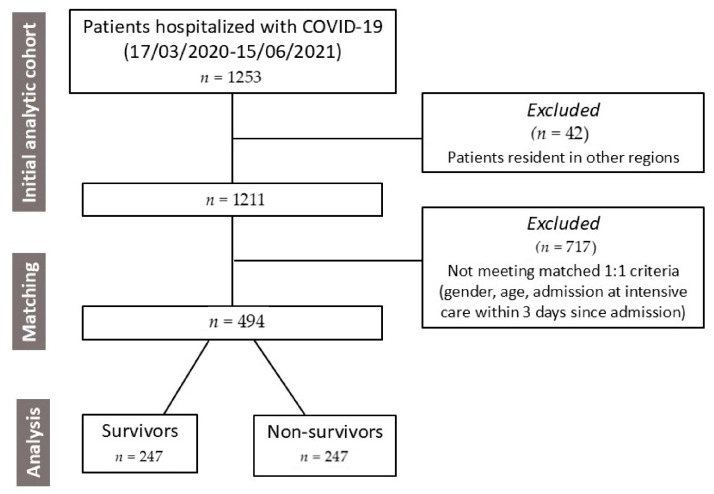
Flow chart illustrating the patients’ selection for this retrospective study.

This observational, matched cohort study was conducted in the Regional General Hospital “F. Miulli” in Acquaviva delle Fonti, Bari, Italy, a 600-bed referral center that dedicated up to 300 beds to COVID-19 patients during pandemic emergency from 17 March 2020 to 15 June 2021. The primary endpoint of this study was the mortality at 90 days in patients admitted for COVID-19. For patients residing in Puglia, deaths occurring after hospital discharge were detected by the regional health information system. Among 1253 patients admitted for COVID-19, 1211 were evaluated for this study after the exclusion of those resident in other regions. Because several baseline variables were different between survivors and non-survivors, we matched the two cohorts (247 survivors and 247 deceased within 90 days from hospitalization) using an automated procedure to select similar patients 1:1 according to gender, age (difference lower than 5 years), admission (difference up to 30 days), and intensive care needs within three days since admission (Figure 3).

The following data of the selected patients were collected: Demographic information (age, gender), clinical symptoms suggestive of COVID-19, comorbidities, vital signs, pharmacological treatments (before and during hospitalization), in-hospital course (admission in intensive care unit and respiratory support measures), complications (ARDS at admission or developed during hospitalization), mortality, and laboratory tests. All data were recorded on an electronic datasheet.

This study was approved by the institutional review board (#7780, 6 June 2023, Independent Ethical Committee “Consorziale Policlinico”—Policlinico of Bari, Italy) and conducted in accordance with the principles of the Declaration of Helsinki [26]. All the data of interest were collected by consulting the patients’ medical records. The study population was divided according to mortality at 90 days to detect the potential protective effects of OAT on that endpoint.

### 2.2. Statistical Analysis

Data are reported as mean ± standard deviation, median (interquartile range), or percentage for categorical variables. Data of the paired patients were compared with a dependent samples *t*-test (continuous variables) and McNemar’s test (dichotomous information). A conditional logistic regression model, appropriate for matched data, was used to compare the data of paired patients. To investigate the association between OAT and mortality at 90 days, an odds ratio (OR) value with a 95% confidence interval (CI) was calculated for the survivors’ and non-survivors’ subgroups encompassing age, gender, comorbidities, clinical and respiratory parameters at admission, and pharmacotherapies. *p* values < 0.05 were considered statistically significant. All analyses were conducted using STATA software, version 16 (Stata-Corp LP, College Station, TX, USA).

## 3. Results

A total of 494 patients were matched 1:1 according to mortality at 90 days after hospital admission, including 247 survivors and 247 non-survivors. In Table 1, patients’ characteristics (i.e., demographics, comorbidities, pharmacotherapies) at hospital admission are summarized. Groups were similar in terms of matching criteria: age (77 ± 10 years), gender (141 men and 106 women), and ICU admission within 3 days (25 patients in each cohort). As can be deduced from the univariate regression analysis (Table 1), mortality at 90 days appeared to be significantly associated with HF (*p* < 0.001), arteriopathy (*p* = 0.007), which includes diverse events (e.g., myocardial infarction and ischemia, cerebrovascular disorders), asthma (*p* = 0.007), AF (*p* = 0.009), histories of cancer (*p* = 0.011), and kidney failure (KF) and COPD as well.

Regarding pre-admission pharmacotherapies, neither DOACs nor warfarin, and heparins as well, provided statistically significant beneficial effects (*p* > 0.05). No significant association with OAT was observed between survivors and dead patients, also adjusting for baseline predictors of mortality (OR 0.85; CI 0.50–1.46, *p* = 0.565). With a few exceptions (ACE inhibitors, antidiabetics, and statins), no pharmacological treatment showed a favorable trend (i.e., higher frequency in survivors); whereas, patients treated with aldosterone antagonists were significantly more frequent (*p* < 0.001) in non-survivors (30.0% vs. 13.8%).

A multivariate analysis of baseline variables (comorbidities and pharmacotherapies at hospital admission) showing significant association (*p* < 0.05) to the endpoint in a univariate analysis, revealed that, compared to those living at 90 days from hospitalization, deceased patients had cancer histories (OR 1.75, CI 1.06–2.90, *p* = 0.029) or suffered from asthma (OR 2.25, CI 1.13–4.47, *p* = 0.021) (Table S1 in Supplementary Materials). In contrast, HF, AF, arteriopathy, COPD, and kidney failure (KF), which, in the univariate analysis, were found to be associated with the endpoint (*p* < 0.05), lost significance in the multivariate analysis. Therapy at admission with aldosterone antagonists (mostly canrenone) was also confirmed to be significantly associated with the endpoint in the multivariate analysis (OR 2.49, CI 1.52–4.08, *p* < 0.001).

As summarized in Table 2, the clinical features of non-survivor patients within 90 days after admission, especially those related to respiratory parameters at admission and respiratory supports, were worse than those of survivors. As for the pharmacological treatments, the data related to the broad-spectrum antiviral remdesivir and the HIV-targeted antiretroviral lopinavir, repurposed against SARS-CoV-2, are too few for drawing conclusions, despite some trends. The same applies to the old antimalarial drug hydroxychloroquine (often administered in combination with the macrolide antibiotic azithromycin), supposedly able to prevent SARS-CoV-2 infection during the first phase of pandemics and later proven to lack clinical benefit for COVID-19 [27,28]. Indeed, despite the statistical significance (*p* = 0.007) of the difference between the survivors’ group (9.7% frequency) and non-survivors (5.3% frequency), the evidence remains inconclusive. A favorable trend might be inferred for azithromycin.

Subjects taking subcutaneous low-molecular-weight heparins (LMWHs) did not experience any significant beneficial effect in terms of 90-day mortality. In contrast, among non-survivors, patients treated with unfractioned heparins (HMWHs) were twice as many as those found among survivors. At admission, patients receiving OAT were 60 (24.3%) in the group of survivors (21 in the DOAC group and 12 in the VKA–warfarin group; 27 started OAT during hospitalization) and 69 (27.9%) in deceased patients (25 in the DOAC group and 13 in the VKA group; 31 started OAT during hospitalization). No other pharmacological treatment proved to significantly affect medium-term mortality, except for patients treated with vitamin D. Interestingly, vitamin D-treated patients populated the group of survivors (42.9%) much more (*p* < 0.001) than the group of non-survivors (27.9%).

As shown in Table 3, which summarizes clinical parameters at admission, deceased patients were characterized by higher levels of azotemia, serum lactate dehydrogenase, and C-reactive protein. Importantly, IL-6 and procalcitonin, markers of a severe infection in progress, and D-dimers, markers of deep vein thrombosis, pulmonary embolism, as well as inflammation and infection, were higher in non-survivors compared to survivors. According to previous studies [1,2], albumin levels and various parameters from a blood count analysis were altered in non-survivors (Table S2 in Supplementary Materials).

**Figure 4:**
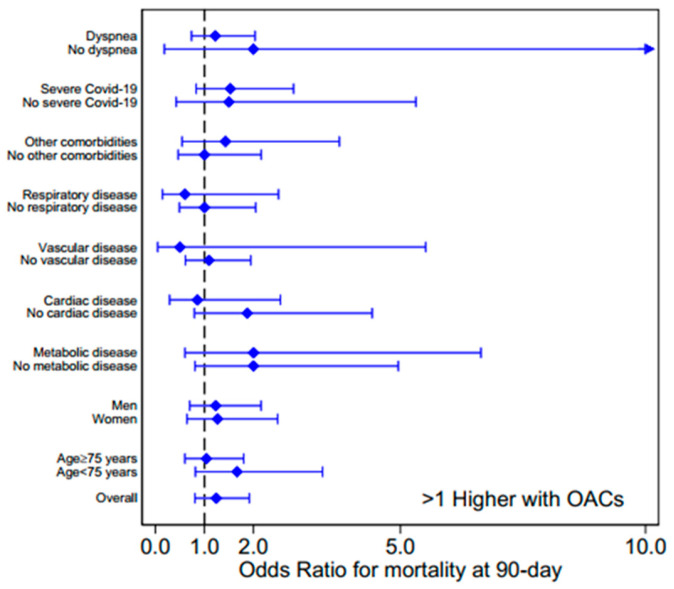
Analysis of odds ratios for subgroups encompassing age, gender, comorbidities, and COVID-19 severity for investigating the association between oral anticoagulant therapy (DOACs or VKA) and mortality. OAC: oral anticoagulant.

The association between OAT (including warfarin as VKA and the DOACs apixaban and edoxaban) and mortality at 90 days was assessed calculating the OR values for the survivors’ and non-survivors’ subgroups encompassing age, gender, comorbidities, clinical and respiratory parameters at admission, and pharmacotherapies (data in Table 1 and Table 2, according to the presence/absence of a categorical factor or by values above/below median for continuous data). The ORs with CI for age, gender, comorbidities, and COVID-19 severity are shown in Figure 4; whereas, the OR subgroup analysis of laboratory parameters and pharmacological therapies, and for clinical and respiratory parameters at admission, are reported in Figures S1 and S2, respectively (Supplementary Materials).

Figure 4 shows that there is no association between OAT and mortality, the OR values being higher than 1, with a large 95% confidence interval, for several conditions (overall OR 1.24, CI 0.81–1.92, *p* = 0.324). Moreover, for each analyzed characteristic and clinical parameter, no difference in OAT prevalence was observed between survivors and dead patients.

## 4. Discussion

The main statistically significant outcomes of this retrospective clinical study are the following: (i) Among the comorbidities, cancers and asthma proved to have the highest negative impact on the medium-term mortality of COVID-19 patients. (ii) OAT (both DOACs and VKAs), either before and during hospitalization, appears not to be significantly associated to beneficial effects. (iii) Aldosterone antagonists in antihypertensive therapy appear to worsen the clinical course of COVID-19 patients; whereas, (iv) vitamin D supplementation appears to have beneficial effects.

### 4.1. Comorbidities Influencing COVID-19-Related Mortality

Cardiovascular comorbidities and complications significantly impact the survival rates of COVID-19 patients [29,30,31]. Therefore, dissecting the intricate relationship between various comorbidities and COVID-19 outcomes can pave the way for developing improved clinical strategies and therapeutic protocols, potentially mitigating the impact of the disease. According to the impact of cardiovascular diseases [32], in our retrospective study, HF, AF, and arteriopathy-related events, characterized by a pro-thrombotic state so that they frequently complicate each other, are assessed by univariate logistic regression as cardiovascular comorbidities associated to the medium-term mortality of our cohort of COVID-19 patients (*p* < 0.05). As reported, thromboembolic complications result in death increase in COVID-19 patients. Although the above diseases lost significance in the multivariate regression analysis (Table S1 in Supplementary Materials), our analysis revealed the impact on the mortality of COVID-19 patients in a blood coagulation state. The high level of D-dimers, indicating an increased hypercoagulation, together with elevated levels of IL-6 and CRP, indicating sustained inflammation, are associated with an increase in infection, sepsis, and mortality in COVID-19 patients [33,34]. As a matter of fact, in our patients’ cohort, D-dimers, IL-6, procalcitonin, and CRP were found to be significantly higher (*p* < 0.001) in non-survivors compared with surviving patients, which highlights the effect of increased inflammation and thromboembolic complications on the medium-term mortality of COVID-19 patients.

The multivariate analysis of comorbidities at hospital admission, confirming the univariate regression results, indicates cancer as the one significantly associated with the medium-term mortality of the examined cohort of COVID-19 patients. As for respiratory diseases, the univariate regression analysis revealed asthma and COPD (that instead lost significance in multivariate significance) as significantly associated with the endpoint. In addition, our study confirms that respiratory impairment has a significant impact on mortality, as indicated by respiratory insufficiency, dyspnea, and high values of positive end expiratory pressure (PEEP), significantly characterizing the population of non-survivors with respect to survivors (*p* < 0.001).

### 4.2. Impact of Oral Anticoagulation Therapy on COVID-19 Clinical Course

The main purpose of this study was to investigate the eventual association between OAT and mortality in COVID-19 patients. An increased number of studies have shown abnormal serum coagulation parameters in hospitalized patients with severe forms of COVID-19 with a trend toward a hypercoagulable state [34,35,36]. Thus, during the pandemic, anticoagulation treatment was considered a pharmacological strategy to reduce the risk of mortality in patients affected by a severe form of COVID-19 [37,38], but the true benefit of OAT on the clinical outcomes of hospitalized patients is still debated.

In our patients’ cohort, OAT with either DOACs or VKAs before and during hospitalization proved to not exert any beneficial effect on mortality within 90 days after SARS-CoV-2 infection. Our findings are consistent with those reported in previous observational studies and meta-analyses [38,39,40]. Although the mechanisms underlying SARS-CoV-2 pathogenesis are not yet completely understood, the interplay between inflammation and coagulation should have a pivotal role. Indeed, the severe inflammatory response and disseminated intravascular coagulation together with virus-induced local inflammatory reactions may affect endothelial cell function leading to vessel wall damage and consequent microvascular thrombosis [7,41,42]. Some authors hypothesized that microvascular pulmonary thrombosis in COVID-19-induced pneumonia is sustained by a complex interplay between clotting system activation and an immune-mediated inflammatory response [43,44]. Based on this mechanism, which involves two processes mutually reinforcing each other, it might be argued that, as likely occurred in the investigated cohort of COVID-19 patients, OAT, by targeting one single pathway, cannot favorably affect the progression of SARS-CoV-2 infection and the natural history of the disease.

Another explanation of the lacking association between OAT and beneficial effect on mortality could be derived from the current view regarding the mechanisms responsible for COVID-19-associated coagulopathy in which fibrin is considered as a key driver of thromboinflammation [45]. Indeed, very recently, it has been demonstrated that the SARS-CoV-2 spike protein interacts with fibrinogen, promoting fibrin polymerization and, ultimately, triggering its thromboinflammatory activity [46]. In this light, we can postulate that, despite OAT, blood clots in the COVID-19 patients’ cohort remain resistant to degradation, since the hypercoagulation state is mainly due to elevated plasma fibrinogen, which is, on the other hand, supported by the elevated D-dimer levels in non-survivors. Interestingly, fibrin-targeted immunotherapy is currently proposed for patients with acute and long COVID-19 [45].

### 4.3. Impact of Other Pharmacological Treatments on COVID-19 Mortality

As highlighted above, the use of the aldosterone antagonist canrenone, i.e., the active metabolite of spironolactone, in the form of canrenoic acid or potassium canrenoate, appeared to worsen the clinical course of the investigated COVID-19 patients’ cohort. This was somehow surprising, due to the potential of MRAs to prevent aldosterone from causing fibrosis and inflammation. Indeed, it has been reported that aldosterone levels increase in COVID-19 patients, and some studies demonstrated that treatment with MRAs has an overall positive impact on clinical improvement and all-cause mortality [47,48,49]. However, a systemic review and meta-analysis did not establish a significant association between MRA therapy and mortality in patients infected with SARS-CoV-2 [50], and the effect of canrenone in COVID-19 patients still remains uncertain.

**Figure 5:**
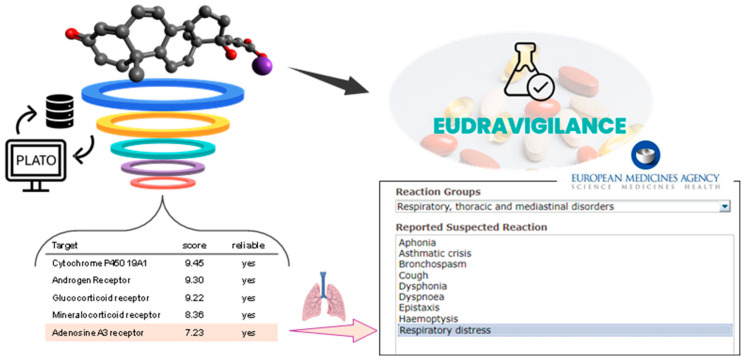
By using the potassium canrenoate as query, PLATO returned the A_3_R as a reliable target. ADR (adverse drug reaction) analysis on Eudravigilance database showed, among others, side effects, specifically at the respiratory tract, associated with potassium canrenoate use.

To possibly understand the canrenone capability of increasing the severity of SARS-CoV-2 infection, maybe postulating undisclosed off-target drug activities, we queried a digital platform, namely PLATO (i.e., polypharmacology platform prediction), developed by some of us for efficient target fishing and the bioactivity profiling of bioactive small molecules [51,52]. The target fishing tool of PLATO (Figure S3 in Supplementary Materials) prioritized, after the expected targets (androgen, glucocorticoid, mineralcorticoid, and progesterone receptors), the A_3_ adenosine receptor (A_3_AR) among the potential targets of canrenone (Figure 5). This in silico outcome was rather interesting, considering that several studies showed that A_3_AR, together with other purinergic receptors, may have a role in lung inflammation and that targeting these receptors for pulmonary diseases might be of therapeutic interest [53,54,55]. As a matter of fact, the modulation of purinergic receptors has been demonstrated to have an impact on the severe consequences of COVID-19 [56,57]. As far as A_3_AR signaling transduction is concerned, its stimulation inhibits neutrophil degranulation in neutrophil-mediated tissue injury, TNFα and platelet activation, and the factor-induced chemotaxis of human eosinophils [54]. Importantly, based on the ability to reduce levels of inflammatory mediators, piclidenoson, an A_3_AR agonist, was tested for compassionate use in COVID-19 patients [54,58,59]. Thus, it can be hypothesized that canrenoate interacting with A_3_AR at pulmonary levels might act as an antagonist, in addition to eliciting other biological responses through its interaction with other steroid receptors, worsening the course of disease. In support of this hypothesis, among the adverse drug reactions reported for potassium canrenoate in the Eudravigilance database [60], there are some concerning the system organ class “Respiratory, thoracic, and mediastinal disorders” with suspected reactions, including bronchospasm, cough, dyspnea, and respiratory distress (Figure 5).

Unlike canrenoate, supplementation with vitamin D proved to be significantly associated with improved clinical outcome of the hospitalized COVID-19 patients, at least with respect to the endpoint of medium-term mortality. Notably, among the survivors, 106 patients (42.9%) were receiving vitamin D, compared to only 69 patients (27.9%) among the deceased ones. This observation should be in line with the established anti-inflammatory properties of vitamin D and its impact on the management of the SARS-CoV-2 infection since the onset of the pandemic. Actually, a large meta-analysis indicates that vitamin D, in its active form, namely calcitriol, may have both beneficial and potentially negative effects in COVID-19 patients [61]. Calcitriol enhances ACE2 expression, helping to restore the ACE2/ACE ratio, which counteracts the pro-inflammatory and pro-thrombotic effects of SARS-CoV-2 [62]. Calcitriol has also been shown to reduce inflammation by inhibiting TLR-2 and the NLRP3 inflammasome, while promoting autophagy and antimicrobial peptide release. However, some studies, including the CORONAVIT trial, suggest that high doses of vitamin D may not effectively reduce the risk of infection or improve outcomes in hospitalized patients [63]. While calcitriol can reduce inflammation and enhance immune responses, it may also favor the production of certain cytokines, complicating its overall effectiveness. Our findings, however, would suggest the beneficial effects of vitamin D supplementation. The apparent discrepancy with literature and clinical data once again underlines the need for further studies investigating the therapeutic implications of vitamin D in the management of COVID-19.

## 5. Conclusions

Our objective was not the identification of the independent predictors of COVID-19 medium-term mortality, but the retrospective evaluation of the eventual beneficial impact of OAT, based on both VKAs and DOACs, on patients’ subgroups encompassing comorbidities and pharmacotherapies at baseline (hospital admission) and during hospitalization. As major outcomes of the study, cancer, which may also be associated with coagulopathy, and asthma, which instead may enhance the severity of COVID-19, proved to be most highly associated with the medium-term mortality of the examined patients’ cohort.

Among the cardiovascular diseases, HF, AF, and arteriopathy in the univariate analysis were found to be associated with the endpoint but lost significance in the multivariate regression analysis. The high level of D-dimers, which relate to hypercoagulation, combined with elevated levels of IL-6 and CRP, which reveals sustained inflammation, is most likely associated with increased infection, sepsis, and mortality in COVID-19 patients. Although there is a significant higher level in non-survivor patients of the biomarkers of the procoagulant state, no statistically significant association was found between OAT, with either warfarin as VKA or apixaban (and edoxaban in a few cases) as DOAC, and the severity of COVID-19 disease and medium-term mortality. Clinical data indicate that there is no evidence of harmful effects of VKA or DOAC (i.e., the number of patients in OAT are practically the same in the surviving and deceased patients’ groups) on the course of COVID-19 disease; although, both drugs, similarly to subcutaneous administered LMWHs, do not alleviate the disease severity.

Although not definitive, two main findings emerge with respect to the other pharmacological treatments: (i) anti-aldosterone drugs taken by hypertensive patients at admission were found to be significantly associated with 90-day mortality; (ii) vitamin D showed benefit against COVID-19. While the effects of vitamin D had been reported in the literature, although with contrasting arguments [61], the negative effects of anti-aldosterone drugs in the examined cohort appear consistent with off-target activities highlighted by the Eudravigilance database and the digital predictive platform PLATO.

## Figures and Tables

**Table 1:** Patients’ characteristics at hospital admission according to 90 day mortality.

Patients’ Characteristics	Survivors	Non-Survivors	*p* Value
	*n* = 247	*n* = 247	
Demographics			
Age (years)	77 ± 10	77 ± 10	-
Males	141 (57.1%)	141 (57.1%)	-
Comorbidities			
Obesity	45 (18.2%)	35 (14.2%)	0.225
Arterial hypertension	195 (78.9%)	191 (77.3%)	0.663
Dyslipidemia	29 (11.7%)	31 (12.6%)	0.782
Diabetes mellitus	67 (27.1%)	82 (33.2%)	0.143
Previous myocardial infarction	21 (8.5%)	28 (11.3%)	0.307
Peripheral vasculopathy	14 (5.7%)	20 (8.1%)	0.289
Arteriopathy	19 (7.7%)	39 (15.8%)	0.007
Stroke or transient ischemic attack	20 (8.1%)	19 (7.7%)	0.869
History of heart failure (HF)	26 (10.5%)	57 (23.1%)	<0.001
History of cancer	38 (15.4%)	61 (24.7%)	0.011
Asthma	20 (8.1%)	39 (15.8%)	0.007
Chronic obstructive pulmonary disease (COPD)	61 (24.7%)	83 (33.6%)	0.038
Atrial fibrillation (AF)	33 (13.4%)	55 (22.3%)	0.009
Chronic liver disease	22 (8.9%)	24 (9.7%)	0.758
Kidney failure (KF)	52 (21.1%)	74 (30.0%)	0.016
Pharmacotherapy at hospital admission			
ACE inhibitors ^a^	76 (30.8%)	58 (23.5%)	0.061
AT2 receptor blockers ^b^	62 (25.1%)	69 (27.9%)	0.490
Antidiabetics ^c^	51 (20.6%)	43 (17.4%)	0.317
Aspirin	55 (22.3%)	74 (30.0%)	0.051
Heparins ^d^	11 (4.5%)	14 (5.7%)	0.664
DOACs ^e^	21 (8.5%)	25 (10.1%)	0.547
VKA (warfarin)	12 (4.9%)	14 (5.7%)	0.839
Vitamin D	7 (2.8%)	17 (6.9%)	0.052
Diuretics ^f^	89 (36.0%)	103 (41.7%)	0.174
Corticosteroids ^g^	21 (8.5%)	27 (10.9%)	0.355
Aldosterone antagonists ^h^	34 (13.8%)	74 (30.0%)	<0.001
Hypocholesterolemic agents ^i^	24 (9.7%)	18 (7.3%)	0.289

^a^ Angiotensin-converting enzyme inhibitors: enalapril, perindopril, ramipril, zofenopril, lisinopril, delapril, and fosinopril. ^b^ Angiotensin II receptor type 2 blockers: valsartan, telmisartan, candesartan, olmesartan, irbesartan, losartan, and association of valsartan with sacubitril (prodrug of inhibitors of the enzyme neprilysin). ^c^ Mostly oral hypoglycemics (metformin, repaglinide, pioglitazone, alogliptin, linagliptin, and empaglifozin), sometimes in combination therapy, and a few subcutaneous insulin treatments. ^d^ Mostly, subcutaneous administered low-molecular-weight heparins (LMWHs) and fondaparinux. ^e^ Mostly, the fXa-selective inhibitor, apixaban, and edoxaban. ^f^ Furosemide, hydrochlorothiazide, acetazolamide, and indapamide. ^g^ Mostly cortisone acetate and prednisone. ^h^ Canrenone, potassium canrenoate, and spironolactone. ^i^ Cholesterol-lowering statins (atorvastatin, rosuvastatin, and simvastatin) and ezetimibe.

**Table 2:** Patients’ clinical presentation, respiratory supports, and pharmacological treatments during hospitalization according to 90-day mortality endpoint.

Clinical Data and Interventions	Survivors	Non-Survivors	*p* Value
	*n* = 247	*n* = 247	
Intensive care within 3 days since admission	25 (10.1%)	25 (10.1%)	-
Systolic blood pressure (mm Hg)	137 ± 22	131 ± 25	0.072
Diastolic blood pressure (mm Hg)	73 ± 13	72 ± 14	0.311
Heart rate (b/min)	80 ± 15	85 ± 17	<0.001
Severe COVID-19	162 (65.6%)	190 (76.9%)	0.003
Respiratory insufficiency	195 (78.9%)	229 (92.7%)	<0.001
Cough	133 (53.8%)	48 (19.4%)	<0.001
Dyspnea	199 (80.6%)	206 (83.4%)	0.406
Fever	134 (54.3%)	101 (40.9%)	0.003
Asthenia	145 (58.7%)	174 (70.4%)	0.006
PEEP (cmH_2_O) ^a^	54 (21.9%)	140 (56.7%)	<0.001
PaO_2_ (mmHg) ^a^	70 ± 23	67 ± 24	0.087
FiO_2_ (%) ^a^	28 ± 16	30 ± 17	0.241
PaO_2__FiO_2_ (mmHg) ^a^	285 ± 105	264 ± 111	0.007
PaCO_2_ (mmHg) ^a^	38 ± 27	35 ± 10	0.542
Respiratory support			
Noninvasive ventilation	39 (15.8%)	86 (34.8%)	<0.001
Mask	146 (59.1%)	103 (41.7%)	<0.001
Mechanical ventilation	26 (10.5%)	84 (34.0%)	<0.001
Low-flow oxygen devices	67 (27.1%)	31 (12.6%)	<0.001
High-flow oxygen devices	131 (53.0%)	200 (81.0%)	<0.001
Pharmacological treatment			
Remdesivir	12 (4.9%)	3 (1.2%)	0.035
Lopinavir	10 (4.0%)	8 (3.2%)	0.727
Hydroxychloroquine	24 (9.7%)	13 (5.3%)	0.007
Azithromycin	106 (42.9%)	87 (35.2%)	0.084
Low-molecular-weight heparins (LMWHs)	176 (71.3%)	160 (64.8%)	0.106
High-molecular-weight heparins (HMWHs)	42 (17.0%)	88 (35.6%)	<0.001
Oral anticoagulant therapy (OAT: DOACs or VKA) ^b^	52 (21.1%)	53 (21.5%)	0.904
Corticosteroids ^c^	179 (72.5%)	202 (81.8%)	0.006
Insulin	71 (28.7%)	95 (38.5%)	0.022
Aspirin	60 (24.3%)	82 (33.2%)	0.026
Statins ^d^	64 (25.9%)	56 (22.7%)	0.404
Vitamin D	106 (42.9%)	69 (27.9%)	<0.001
Vitamin C	30 (12.1%)	38 (15.4%)	0.194

^a^ PEEP, positive end expiratory pressure; PaO_2_, partial pressure of arterial oxygen; FiO_2_, fraction of inspired oxygen; PaCO_2_, partial pressure of arterial carbon dioxide. ^b^ DOACs (apixaban and edoxaban) and VKA (warfarin) evaluated together. ^c^ Cortisone acetate and prednisone. ^d^ Atorvastatin and rosuvastatin.

**Table 3:** Patients’ laboratory data at hospital admission according to 90-day mortality.

Laboratory Data	Survivors	Non-Survivors	*p* Value
	*n* = 247	*n* = 247	
Azotemia	62 ± 40	82 ± 51	<0.001
Estimated glomerular filtration rate (eGFR)	67 ± 29	58 ± 30	0.001
Potassium (mmol/L)	4.18 ± 0.68	4.10 ± 0.68	0.179
Sodium (mmol/L)	139 ± 5	140 ± 7	0.212
Lactate dehydrogenase (U/L)	323 ± 152	393 ± 250	<0.001
Lipase (U/L)	215 ± 214	218 ± 370	0.225
C-reactive protein (CRP, mg/L)	7 ± 6	10 ± 7	<0.001
Pro-calcitonin (ng/mL)	0.6 ± 4.2	0.8 ± 2.0	<0.001
Erythrocyte sedimentation rate (mm/h)	54 ± 30	58 ± 27	0.287
Interleukin 6 (pg/mL)	47 ± 50	141 ± 539	<0.001
Prothrombin time INR	1.29 ± 0.73	1.23 ± 0.46	0.261
Prothrombin time Ratio	1.28 ± 0.70	1.23 ± 0.44	0.258
Vitamin 25-OH-D3 (ng/mL)	16 ± 10	19 ± 16	0.128
Activated partial thromboplastin time ratio	1.02 ± 0.23	1.15 ± 0.64	0.081
Calcium (mg/dL)	8.47 ± 0.61	8.35 ± 0.82	0.016
Amylase (U/L)	67 ± 46	68 ± 63	0.228
Direct bilirubin (mg/dL)	0.23 ± 0.18	0.38 ± 0.96	0.025
Total bilirubin (mg/dL)	0.63 ± 0.46	0.80 ± 1.22	0.208
D-dimers (ng/mL)	3427 ± 9634	6227 ± 18,858	<0.001
Ferritin (ng/mL)	958 ± 2853	1197 ± 2313	0.007
Gamma-GT (U/L)	58 ± 82	88 ± 158	0.132
Glycemia (mg/dL)	130 ± 70	136 ± 63	0.199

## Data Availability

Data available upon request.

## Supplementary Materials

The following supporting information can be downloaded at: https://www.mdpi.com/article/10.3390/biomedicines13030535/s1, Figure S1: Analysis of Odds Ratios for subgroups encompassing laboratory parameters and pharmacological therapies at admission for investigating the association between oral anticoagulant therapy and mortality; Figure S2: Analysis of Odds Ratios for subgroups encompassing clinical and respiratory parameters at admission for the association between oral anticoagulant therapy and mortality; Figure S3: Output report from target fishing analysis of potassium canrenoate by PLATO-Polypharmacology pLATform prediction (https://prometheus.farmacia.uniba.it/plato/, accessed on 18 December 2024); Table S1: Multivariate analysis of clinical variables (comorbidities, pharmacotherapies) of COVID-19 patients at hospital admission according to 90-day mortality endpoint; Table S2: Laboratory data of blood count and serum proteins of patients at hospital admission according to 90-day mortality.
