# Supplementary material for: Retrospective Clinical Investigation into the Association Between Abnormal Blood Clotting, Oral Anticoagulant Therapy, and Medium-Term Mortality in a Cohort of COVID-19 Patients
Source: Biomedicines. 2025 Feb 20;13(3):535. doi: 10.3390/biomedicines13030535 (PMC11940371; doi:10.3390/biomedicines13030535)
Supplement: Supplementary file 1 [file biomedicines-13-00535-s001.zip › biomedicines-3408040-supplementary.pdf]

Retrospective Clinical Investigation into the Association Between Abnormal Blood Clotting, Oral Anticoagulant Therapy, and Medium-Term Mortality in a Cohort of COVID-19 Patients

Giorgia Dinoi [1,†], Maria Vittoria Togo [1,†], Pietro Guida [2], Caterina Deruvo [1], Francesco Samarelli [1], Paola Imbrici [1], Orazio Nicolotti [1], Annamaria De Luca [1], Franco Mastroianni [2], Antonella Liantonio [1,*] and Cosimo Damiano Altomare [1,*]

1 Department of Pharmacy—Pharmaceutical Sciences, University of Bari Aldo Moro, 70125 Bari, Italy; giorgia.dinoi@uniba.it (G.D.); maria.togo@uniba.it (M.V.T.); caterina.deruvo@uniba.it (C.D.); francesco.samarelli@uniba.it (F.S.); paola.imbrici@uniba.it (P.I.); orazio.nicolotti@uniba.it (O.N.); annamaria.deluca@uniba.it (A.D.L.)
2 Department of Internal Medicine, F. Miulli General Hospital, 70021 Bari, Italy; p.guida@miulli.it (P.G.); f.mastroianni@miulli.it (F.M.)
* Correspondence: antonella.liantonio@uniba.it (A.L.); cosimodamiano.altomare@uniba.it (C.D.A.); Tel.: +39-080-544-2793 (A.L.); +39-080-544-2781 (C.D.A.)
† These authors contributed equally to this work.

SUPPLEMENTARY MATERIALS

Figure S1 p. 2
Figure S2 p. 2
Figure S3 p. 3
Table S1 p. 4
Table S2 p. 5

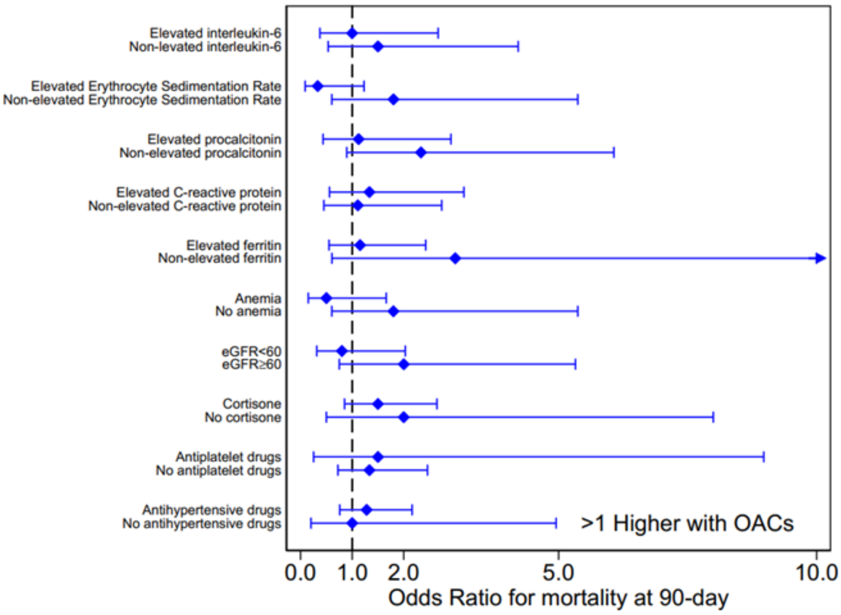

Figure S1. Analysis of Odds Ratios for subgroups encompassing laboratory parameters and pharmacological therapies at admission for investigating the association between oral anticoagulant therapy and mortality.

OAC, oral anticoagulant (DOAC or VKA); **eGFR**, estimated glomerular filtration rate; **Cortisone** stands for anti-inflammatory corticosteroids (cortisone acetate, prednisone); **Antiplatelet drugs**: acetylsalicylic acid (ASA), clopidogrel; **Antihypertensive drugs**: Beta blockers (bisoprolol, nebivolol, atenolol, metopropol, carvedilol, propranolol), calcium channel blockers (nifedipin, amlodipine, verapamil).

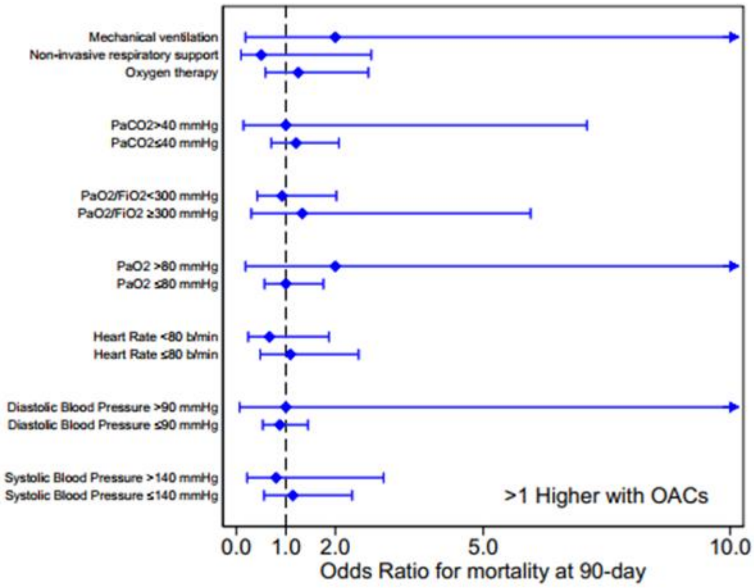

Figure S2. Analysis of Odds Ratios for subgroups encompassing clinical and respiratory parameters at admission for the association between oral anticoagulant therapy and mortality.

OAC, oral anticoagulant; **FiO_2**, fraction of inspired oxygen; **$PaCO_2$**, partial pressure of arterial carbon dioxide; **PaO_2**, partial pressure of arterial oxygen.

Target fishing

smiles: CC12CCC(=O)C=C1C=CC1C2CCC2(C)C1CCC2(O)CCC(=O)[O-].[K+]

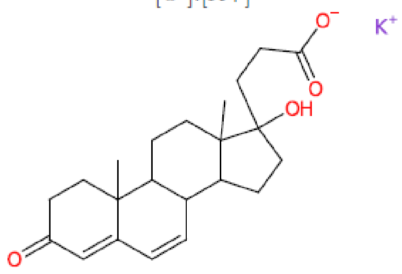

Target	score	reliable
Cytochrome P450 19A1:Homo sapiens	9.45	yes
Androgen Receptor:Homo sapiens	9.30	yes
Glucocorticoid receptor:Homo sapiens	9.22	yes
Androgen Receptor:Rattus norvegicus	9.07	yes
Mineralocorticoid receptor:Homo sapiens	8.36	yes
Testis-specific androgen-binding protein:Homo sapiens	8.23	yes
Tyrosinase:Agaricus bisporus	8.23	yes
Progesterone receptor:Bos taurus	8.11	yes
Corticosteroid binding globulin:Homo sapiens	8.01	yes
Progesterone receptor:Homo sapiens	7.61	yes
Adenosine A3 receptor:Homo sapiens	7.23	yes
Dopamine transporter:Homo sapiens	7.18	yes
MAP kinase ERK1:Homo sapiens	7.13	yes
Constitutive androstane receptor:Mus musculus	7.02	yes
Estrogen receptor beta:Homo sapiens	6.68	yes
Estrogen receptor alpha:Homo sapiens	6.67	yes
Cytochrome P450 2C19:Homo sapiens	6.57	yes
Bile salt export pump:Homo sapiens	6.33	yes
Steroid 5-alpha-reductase 2:Homo sapiens	5.66	no
Niemann-Pick C1-like protein 1:Homo sapiens	5.54	no
Acetylcholinesterase:Homo sapiens	5.53	no
11-beta-hydroxysteroid dehydrogenase 1:Mus musculus	5.52	no
Protein-tyrosine phosphatase 1B:Homo sapiens	5.47	no
Testis-specific androgen-binding protein:Rattus norvegicus	5.42	no
11-beta-hydroxysteroid dehydrogenase 1:Homo sapiens	5.39	no
G-protein coupled bile acid receptor 1:Homo sapiens	5.37	no
11-beta-hydroxysteroid dehydrogenase 2:Homo sapiens	5.34	no
Cytochrome P450 3A4:Homo sapiens	5.29	no
Solute carrier organic anion transporter family member 1A1:Rattus norvegicus	5.27	no
Sigma opioid receptor:Homo sapiens	5.27	no

Figure S3. Output report from target fishing analysis of potassium canrenoate by PLATO - Polypharmacology pLATform prediction (https://prometheus.farmacia.uniba.it/plato/)

Table S1. Multivariate analysis of clinical variables (comorbidities, pharmacotherapies) of COVID-19 patients at hospital admission according to 90-day mortality endpoint.

**Clinical variables** [a]	**OR** [b]	**CI** [b]	***P* value**
Arteriopathy	1.88	0.99-3.57	0.054
History of heart failure	1.68	0.92-3.08	0.090
History of cancer	1.75	1.06-2.90	**0.029**
Asthma	2.25	1.13-4.47	**0.021**
Chronic obstructive pulmonary disease	1.08	0.69-1.70	0.745
Atrial fibrillation	1.39	0.78-2.50	0.268
Kidney failure	1.31	0.79-2.18	0.300
Aldosterone antagonists	2.49	1.52-4.08	**< 0.001**
DOACs	0.85	0.50-1.46	0.565

[a] With the exception of DOACs, only clinical variables with established association with a *P* value < 0.05 from univariate statistical analysis were included in the multivariate analysis. [b] OR: Odds ratio; CI: 95% confidence interval.

Table S2. Laboratory data of blood count and serum proteins of patients at hospital admission according to 90-day mortality.

	Survivors	Non-survivors	*P* value
	n = 247	n = 247	
Blood Count			
Basophiles (10^3/μL)	0.021±0.024	0.023±0.031	0.912
Basophiles (%)	0.28±0.26	0.25±0.27	0.031
Eosinophilic (10^3/μL)	0.032±0.068	0.026±0.073	0.017
Eosinophilic (%)	0.50±1.08	0.29±0.72	0.003
Hematocrit (%)	39±6	38±7	0.040
Hemoglobin (g/dL)	12.7±2.2	12.3±2.2	0.008
Lymphocytes (10^3/μL)	1.08±0.91	0.98±0.95	0.008
Lymphocytes (%)	16±10	13±10	< 0.001
Mean Corpuscular Hemoglobin (pg)	29.1±3.4	28.8±3.2	0.287
Mean Corpuscular Hemoglobin Conc (g/dL)	32.8±1.3	32.4±1.6	0.007
Mean Cell Volume (fL)	89±9	89±9	0.604
Monocytes (10^3/μL)	0.57±0.56	0.59±0.88	0.721
Monocytes (%)	8±5	7±7	< 0.001
Neutrophils (10^3/μL)	5.7±3.3	7.6±4.7	< 0.001
Neutrophils (%)	75±13	80±13	< 0.001
Platelets (10^3/μL)	220±94	218±99	0.909
Red Blood Cells (10^6/μL)	4.41±0.81	4.29±0.82	0.039
Red Cell Distribution Width (%)	14.4±2.5	15.2±3.1	< 0.001
Red Cell Distribution Width (fl)	46±8	49±9	< 0.001
White Blood Cells (10^3/μL)	7.4±3.6	9.2±5.1	< 0.001
Serum Proteins			
Albumin (g/dL)	48±5	46±6	< 0.001
Alpha-1 (g/dL)	7.2±1.6	8.3±2.1	< 0.001
Alpha-2 (g/dL)	15.6±3.0	16.0±3.3	0.503
Beta-1 (g/dL)	6.09±0.78	6.04±0.90	0.557
Beta-2 (g/dL)	6.2±1.1	6.8±1.8	0.006
Gamma (g/dL)	16.5±3.8	16.8±4.7	0.784
Albumin/Globulin	0.96±0.20	0.88±0.20	< 0.001
